# Malignant pleural effusion due to metastatic squamous cell carcinoma exhibiting pseudoglandular and signet ring-like features: a case report

**DOI:** 10.1186/s13256-025-05510-w

**Published:** 2025-12-05

**Authors:** Qing Yu, Zhirong Yang

**Affiliations:** Department of Pathology, Deyang People’s Hospital, 173 Taishan North Road (Section 1), Deyang, Sichuan Province, 618000 China

**Keywords:** Case report, Squamous cell carcinoma, Serous fluid cytopathology, Pseudoglandular features, Signet ring-like features

## Abstract

**Background:**

Malignant pleural effusion due to metastatic squamous cell carcinoma is uncommon. Keratinizing squamous cell carcinoma is morphologically identifiable, whereas nonkeratinizing squamous cell carcinoma poses diagnostic challenges and is prone to underdiagnosis or misdiagnosis.

**Case presentation:**

This study reports the case of a 66-year-old East Asian man with a history of esophageal squamous cell carcinoma who developed pleural metastasis 3 years after chemotherapy, presenting with persistent, dull right-sided chest pain of unknown origin. Chest computed tomography revealed a large left pleural effusion with partial atelectasis. Approximately 600 ml of bloody fluid was drained via thoracentesis and catheter drainage. Cytological examination showed tumor cells exhibiting ring-like and pseudoglandular structure. The final cytological diagnosis was metastatic squamous cell carcinoma within the pleural effusion. The patient opted for symptomatic and conservative management and died 9 months after malignant pleural effusion diagnosis during follow-up.

**Conclusion:**

When metastatic squamous cell carcinoma in pleural effusion is poorly differentiated and exhibits atypical changes such as pseudoglandular and signet ring-like features, vigilance is required during serous fluid cytopathological diagnosis. An accurate diagnosis provides the basis for subsequent clinical treatment.

## Background

Malignant pleural effusion (MPE) is an increasingly prevalent complication in oncological patients, negatively impacting their quality of life and prognosis. Diagnosis typically involves ultrasound-guided pleural effusion for cytological examination [1]. The most common primary tumors causing MPE are lung adenocarcinoma, breast cancer, and ovarian cancer [2]. MPE due to metastatic squamous cell carcinoma (SCC) is rare, particularly when the SCC is poorly differentiated. Diagnosis is challenging owing to the lack of typical keratinized cytoplasm and the presence of atypical changes, such as pseudoglandular and signet ring-like features. Herein, we report a case of metastatic esophageal SCC exhibiting these atypical features in pleural fluid 3 years after the initial diagnosis.

## Case presentation

On 1 August 2022, a 66-year-old East Asian male with a 30-pack-year smoking history was admitted to our hospital with a persistent, dull right-sided chest pain of 2 weeks’ duration without an obvious cause. Admission chest computed tomography (CT) revealed a large left pleural effusion with partial atelectasis.

In 2015, the patient underwent surgery for “benign prostatic hyperplasia” at Huaxi Hospital; however, histopathological examination revealed prostate adenocarcinoma (Gleason score 4 + 5 = 9, International Society of Urological Pathology/World Health Organization (ISUP/WHO) 2016 grade group 5). No adjuvant radiotherapy or chemotherapy was administered. In June 2019, he presented with progressive dysphagia and was diagnosed with stage IV nonkeratinizing SCC of the lower esophagus, metastatic to bilateral supraclavicular, mediastinal, hilar, axillary, porta hepatis, and retroperitoneal lymph nodes. No unusual morphological patterns (e.g., signet ring-like or pseudoglandular differentiation) were observed in the initial tumor specimens. From June to October 2019, he completed six cycles of radical TP regimen chemotherapy (paclitaxel 220 mg IV day 1, nedaplatin 220 mg IV day 2) concurrent with intensity-modulated radiotherapy for esophageal cancer (total dose DT 50 Gy/25F). In July 2021 (over a year prior to admission), he underwent a right orchiectomy for a testicular mass. Pathology revealed metastatic prostate acinar adenocarcinoma (immunohistochemistry: CK +, P504S +, AR (androgen receptor, partially +), paired box gene (PAX)-8-, PAX-2-, MelanA-). The patient has no family history of malignancy in first-degree relatives and is a retired factory worker with limited social support.

Dullness to percussion and diminished breath sounds over the right lower lung field were observed. Firm, fixed, nontender bilateral cervical, supraclavicular, and axillary lymphadenopathy was noted. No palpable abdominal masses were identified, as well as no jaundice or edema. Signs of cachexia were noted. The patient underwent left thoracentesis with tube placement, draining approximately 600 ml of bloody fluid for cytological examination.

**Fig. 1:**
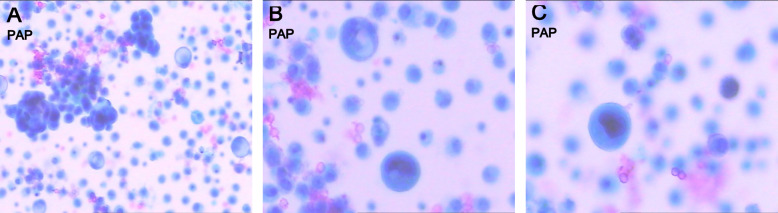
Liquid-based cytology examination of the pleural effusion (Papanicolaou stain). **A** Tumor cells arranged in nests (200 ×). **B** Tumor cells containing cytoplasmic vacuoles resembling signet ring cells (arrow) (400 ×). **C** Scattered tumor cells with intensely hyperchromatic nuclei (“ink-blot” nuclei) (400 ×)

**Fig. 2:**
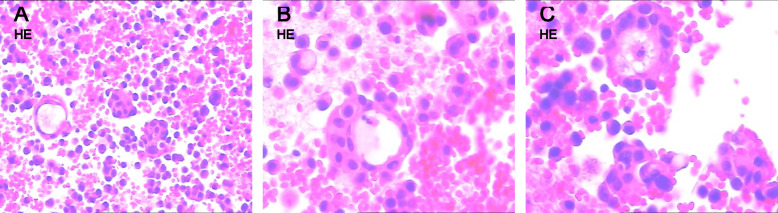
Cell block cytology sections of pleural effusion (Hematoxylin and eosin stain). **A** Tumor cells scattered singly and in clusters (200 ×). **B** and **C** Tumor cells forming pseudoglandular structures (400 ×)

**Fig. 3:**
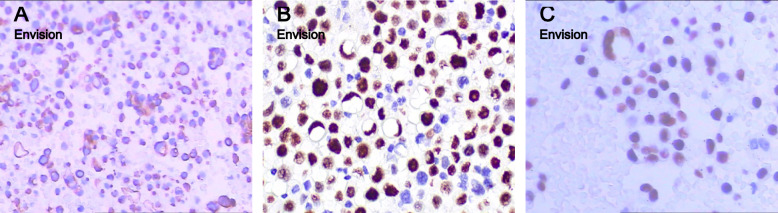
Immunocytochemistry on cell block sections. **A** Pan-cytokeratin positive in the tumor cells (200 ×). **B** P40 positive in tumor cell nuclei (400 ×). **C** P63 positive in tumor cell nuclei (400 ×)

Liquid-based cytology: revealed tumor cells arranged in nests with small nucleoli visible. Some cells contained cytoplasm vacuoles, resembling signet ring cells (Fig. 1a, b). Scattered single cells showed intensely hyperchromatic nuclei (“ink-blot” nuclei) (Fig. 1c). Cell block cytology: revealed round tumor cells scattered singly or in clusters (Fig. 2a). The nuclear-to-cytoplasmic ratio was approximately 1:1, with irregular nuclear membranes and hyperchromasia. Pseudoglandular structures surrounded by tumor cells were observed in some areas (Fig. 2b, c). Immunocytochemical (cell block): pan-cytokeratin (Pan-CK) (+) (Fig. 3a), P40(+) (Fig. 3b), P63(+) (Fig. 3c), androgen receptor(AR)(−), CK8/18(−), P504S (−), prostate-specific antigen(PSA) (−), Napsin A (−), TTF-1 (−), Calretinin (CR)(−). Diagnosis: cytological examination of the pleural effusion identified malignant tumor cells. Combined with the patient’s history and immunocytochemistry profile (P40 +, P63 +, Pan-CK +, negative for adenocarcinoma markers), the findings supported a diagnosis of metastatic esophageal SCC. Clinical course: based on comprehensive findings and American Joint Committee on Cancer (AJCC) 8th Edition criteria, the patient’s TNM (Tumor, Node, Metastasis) stage at MPE diagnosis (August 2022) was cTx N3 M1a | stage IVB esophageal SCC. The cytologically and immunophenotypically confirmed MPE established the M1a status. Following MPE diagnosis, the patient’s Eastern Cooperative Oncology Group (ECOG) performance status declined from 1 to 3 within 6 months. Recurrent dyspnea necessitated therapeutic thoracenteses every 2 weeks for symptom control. Palliative care, including opioid analgesia, was initiated 4 months post-MPE diagnosis, and the patient died 9 months later (May 2023) (Table 1).

**Table 1:** Clinical timeline

Timeline event	Date/timeframe
Esophageal SCC diagnosis	June 2019 (T0)
Completion of chemoradiation	October 2019
Right orchiectomy	July 2021
Pleural effusion onset (Admission)	August 1, 2022 (T1)
Death	May 2023 (T1 + 9mo)

## Discussion and conclusion

This case illustrates the diagnostic challenges posed by metastatic SCC exhibiting unusual cytomorphology in pleural effusion. MPE is a common complication of advanced malignancies, often related to pulmonary metastasis or direct pleural invasion by thoracic tumors. Malignancies from any site can metastasize to the pleura, typically presenting at late stages where biopsy specimens are difficult to obtain. SCC in malignant effusions commonly originates from the lung, female genital tract, or oropharynx. While all types of SCC can involve the pleura, nonkeratinizing SCC is the most prevalent [2]. Morphological identification of keratinizing SCC cells in MPE is usually straightforward. However, diagnosing nonkeratinizing SCC in MPE is challenging and prone to misdiagnosis [3]. The SCC in the patient’s pleural effusion was poorly differentiated and originated from the esophagus. Cytologically, the carcinoma cells lacked characteristic keratinized cytoplasm, consistent with nonkeratinizing SCC. Notably, they exhibited atypical features, including pseudoglandular and signet ring-like morphology. The patient was diagnosed with metastatic esophageal SCC 3 years prior and treated with concurrent chemoradiotherapy. Reactive atypical changes in carcinoma cells can occur following radiotherapy for esophageal cancer [4]. The ring-shaped and pseudoglandular features observed in this patient’s pleural fluid carcinoma cells may be associated with such post-treatment changes.

Given the patient’s history of metastatic prostate adenocarcinoma and the cytomorphologic findings (pseudoglandular structures, signet ring-like features), the differential diagnoses included: (1) metastatic prostate adenocarcinoma—though the patient had prior testicular metastasis, pleural effusion cell block negativity for prostate-specific markers (P504S, PSA) excluded this possibility; (2) metastatic pulmonary adenocarcinoma or squamous cell carcinoma (SCC)—while lung cancer is the most common cause of malignant pleural effusion [5], pulmonary adenocarcinoma was excluded by negative TTF-1/Napsin A staining, and primary lung SCC was ruled out by absent pulmonary lesions on CT; (3) reactive mesothelial cells or mesothelioma—despite nested tumor cell arrangements with peripheral “mulberry” morphology, immunohistochemistry (P40 +, P63 +, CR −) confirmed squamous epithelial origin and negated mesothelial differentiation.

The observed coexpression of pseudoglandular architectures and signet ring-like cytology represents a rarely documented phenotype in metastatic esophageal squamous cell carcinoma (SCC). Contemporary cytopathology literature (2020–2023) reports merely five cases of effusion-based SCC exhibiting both glandular and signet features—all originating from pulmonary primaries [6]. For stage IVB esophageal SCC with malignant pleural effusion (MPE), recent phase II trials indicate promising therapeutic strategies: immune checkpoint/vascular endothelial growth factor (VEGF) inhibition (e.g., sintilimab + anlotinib [7]) achieved 68% MPE control (versus 28% in paclitaxel/nedaplatin historical controls [8]; *p* = 0.002) with median overall survival (OS) of 11.2 months (hazard ratio (HR) 0.62; 95% confidence interval (CI) 0.41–0.93), while cytotoxic T-lymphocyte antigen 4 (CTLA-4)/poly (ADP-ribose) polymerase (PARP) inhibition (tremelimumab + olaparib [9]) demonstrated 52% MPE control and median OS of 9.1 months. Although our patient declined systemic therapy, these regimens represent viable alternatives to palliative drainage alone. Prognostically, MPE cytomorphology informs risk stratification—signet-ring features confer 3.2-fold increased pleural recurrence risk postdrainage (95% CI 1.7–5.9; *p* < 0.01) [10], underscoring the clinical relevance of cytologic subtyping.

This case highlights systemic barriers in real-world diagnostics, particularly for patients with lower socioeconomic status (e.g., retired factory workers with limited social support). Financial obstacles—notably out-of-pocket costs for diagnostic tests (thoracenteses, advanced imaging, immunohistochemical (IHC) panels)—frequently delay diagnosis in resource-limited settings. Health literacy gaps further complicate care, as explaining complex pathology findings (e.g., pseudoglandular versus true glandular morphology) requires simplified communication to ensure informed consent. Delayed referrals also pose significant challenges: nonspecific symptoms (e.g., chest pain) may be misattributed to aging or benign conditions in primary care, postponing specialist evaluation—a recognized failure in fragmented healthcare systems. Implementing cost-reduction policies, patient navigation programs, and standardized referral protocols could mitigate these diagnostic inequities.

Metastatic SCC presenting as MPE is relatively uncommon. This patient, diagnosed with esophageal SCC 3 years prior, developed MPE exhibiting poorly differentiated carcinoma cells with atypical pseudoglandular and signet ring-like features. Such cases necessitate diagnostic vigilance. A thorough patient history and appropriate ancillary studies, particularly immunocytochemistry, are essential for accurate diagnosis and guiding subsequent management.

## Data Availability

No datasets were generated or analyzed during the current study.
